# Mechanisms of Ascorbyl Radical Formation in Human Platelet-Rich Plasma

**DOI:** 10.1155/2014/614506

**Published:** 2014-02-17

**Authors:** Kou-Gi Shyu, Chao-Chien Chang, Yu-Chieh Yeh, Joen-Rong Sheu, Duen-Suey Chou

**Affiliations:** ^1^Division of Cardiology, Shin Kong Wu Ho-Su Memorial Hospital, 95 Wenchang Road, Shihlin Taipei 111, Taiwan; ^2^Graduate Institute of Clinical Medicine, Taipei Medical University, 250 Wu-Hsing Street, Taipei 110, Taiwan; ^3^Department of Cardiology, Cathay General Hospital, 280 Jen Ai Road, Section 4, Taipei 106, Taiwan; ^4^Graduate Institute of Medical Sciences, Taipei Medical University, 250 Wu-Hsing Street, Taipei 110, Taiwan; ^5^Department of Pharmacology, Taipei Medical University, 250 Wu-Hsing Street, Taipei 110, Taiwan

## Abstract

Recently, many clinical reports have suggested that the ascorbyl free radical (Asc^∙^) can be treated as a noninvasive, reliable, real-time marker of oxidative stress, but its generation mechanisms in human blood have rarely been discussed. In this study, we used upstream substances, enzyme inhibitors, and free radical scavengers to delineate the mechanisms of Asc^∙^ formation in human platelet-rich plasma (PRP). Our results show that the doublet signal was detected in PRP samples by using electron spin resonance, and the hyperfine splitting of the doublet signal was *a* ^H^ = 1.88 gauss and *g*-factor = 2.00627, which was determined to be the Asc^∙^. We observed that the inhibitors of NADPH oxidase (NOX), cyclooxygenase (COX), lipoxygenase (LOX), cytochrome P450 (CYP450), mitochondria complex III, and nitric oxide synthase (NOS), but not xanthine oxidase, diminished the intensity of the Asc^∙^ signal dose dependently. All enzyme inhibitors showed no obvious antioxidant activity during a Fenton reaction assay. In summary, the obtained data suggest that Asc^∙^ formation is associated with NOX, COX, LOX, CYP450, eNOS, and mitochondria in human PRP.

## 1. Introduction

Interest in treating oxidative stress has grown in medicine over the past 2 decades. The oxidative status of a biosystem represents a relative level of oxidation in living organisms and is crucial for understanding numerous human physiological and pathophysiological processes [1]. Overproduction of ROS results in oxidative stress, a pathophysiological process that can damage cell structures and induce cancer, cardiovascular disease, atherosclerosis, hypertension, diabetes mellitus, neurodegenerative diseases, rheumatoid arthritis, and ageing. In contrast, ROS play a physiological role in protection against infectious organisms, in the function of several cellular signalling pathways, and the generation of a mitogenic response at low/moderate concentrations [1]. Oxidative status can be estimated using biochemical assays, such as the 2,2′-azino-bis(3-ethylbenzthiazoline-6-sulphonic acid) assay [2], and by measuring the activity of superoxide dismutating enzymes (Mn superoxide dismutase (SOD) and CuZnSOD), catalase (CAT), GSH peroxidase, and reductase [3–5], as well as the level of S-glutathionylation [6]. However, Spasojević suggested that these techniques be supplemented by electron spin resonance (ESR) spectroscopy to enable acquiring data on oxidative status that are more specific [7]. Certain endogenous paramagnetic molecules, such as the ascorbyl free radical (Asc^∙^), tocopheryl radical, and melanin radical, are biomarkers of oxidative status that can be detected using ESR spectroscopy [7].

Ascorbic acid is an essential biological component that can be oxidized through a two-step oxidation process involving a free radical intermediate; this oxidation process may be performed by nearly all oxidizing species intrinsic to the biological environment [7]. When using ESR spectroscopy, the concentration of the Asc^∙^ can be measured using a lower limit of approximately 5 nM with a standard deviation of <1 nM [8]. The characteristics of the Asc^∙^ are relatively stable and it has a long half-life, indicating that it is the most useful biomarker of oxidative status in living systems [7].

ESR spectroscopy was first applied in detecting the Asc^∙^ in oxidative status research in 1993 [9]. Thus far, the Asc^∙^ has been treated as a noninvasive, reliable, real-time biomarker of oxidative stress in various biological samples including plasma, serum, whole blood, cerebrospinal fluid, extracellular fluid, synovial fluid, seminal fluid, tumor, and heart tissue samples [7]; however, the generation mechanisms of the Asc^∙^ in human blood have rarely been discussed.

Ascorbate (the reduced form of vitamin C) is an important radical scavenger and antioxidant in human plasma. Asc^∙^ has been detected by ESR in various biological samples including plasma, serum, whole blood, cerebrospinal fluid, skin, extracellular fluid, synovial fluid, gastric mucosa, seminal fluid, tumors, heart tissue, and others [7]. We recently applied ESR spectroscopy in detecting the Asc^∙^ to investigate the mechanisms of oxidative stress caused by lymphedema in mice [10]. In this study, we used upstream substances, enzyme inhibitors, and free radical scavengers to delineate the mechanisms of Asc^∙^ formation in human platelet-rich plasma (PRP).

## 2. Materials and Methods

### 2.1. Materials

AA861, allopurinol, antimycin, arachidonic acid (AA), baicalein, CAT, clotrimazole, dimethyl sulfoxide (DMSO), diphenyleneiodonium (DPI), ethylenediaminetetraacetic acid, hemoglobin, indomethacin, NG-nitro-L-arginine methyl ester (L-NAME), quinacrine, and SOD were purchased from Sigma Chemical (St. Louis, MO, USA). L(+)-ascorbic acid was purchased from Wako Pure Chemical Industries (Osaka, Japan).

### 2.2. Human Blood Collection Procedure

This study was approved by the Institutional Review Board of Taipei Medical University and conformed to the principles outlined in the Helsinki Declaration. All human volunteers provided informed consent to participate.

### 2.3. Preparation of Human Blood Components

Whole blood was collected from healthy human volunteers who had taken no medicine during the preceding 2 wk and was mixed with acid/citrate/glucose. After centrifugation at 120 ×g for 10 min at room temperature, the supernatant (PRP) was supplemented with PGE_1_ (0.5 *μ*M) and heparin (6.4 IU/mL) and then incubated for 10 min at 30°C and centrifuged at 500 ×g for 10 min. The supernatant was platelet-poor plasma (PPP) and was used in subsequent experiments.

### 2.4. Isolation of Red Blood Cells

Whole blood was centrifuged at 650 ×g for 5 min. Plasma was removed carefully and the white buffy layer was completely removed through aspiration using a pipette with utmost care. The red blood cells (RBCs) were then washed three additional times with Tyrode's solution.

### 2.5. Measurement of the Ascorbyl Free Radical in Platelet-Rich Plasma Using Electron Paramagnetic Resonance Spectrometry

The ESR method involved using a Bruker EMX ESR spectrometer (Bruker Instruments Inc., Billerica, MA, USA) as described previously [11]. The PRP was prewarmed to 37°C for 2 min, and enzyme inhibitors or other reagents were then added. ESR spectra were recorded at room temperature by using a quartz flat cell designed for aqueous solutions. ESR spectrometry was conducted under the following conditions: 20 mW of power at 9.78 GHz, with a scan range of 100 G and a receiver gain of 5 × 10^4^. The modulation amplitude, sweep time, and time constant are provided in the figure legends.

### 2.6. Fenton Reaction Model System with Electron Paramagnetic Resonance Detection of the Hydroxyl Radical

The hydroxyl radical generated in a standard Fenton reaction was trapped using DMPO according to the method previously described [12]. A Fenton reaction solution (50 *μ*M FeSO_4_ + 500 *μ*M H_2_O_2_) was pretreated with a solvent control (0.6% DMSO) or reagent (10 *μ*M). The ESR spectra were recorded after precisely 3 min.

### 2.7. Statistical Analysis

The experimental results are expressed as the mean ± SEM and are accompanied by the number (*n*) of observations. The data were assessed using an analysis of variance (ANOVA). When this analysis indicated significant differences among the group means, each group was compared using the Newman-Keuls method. A *P* value <0.05 was considered statistically significant.

## 3. Results

### 3.1. Electron Spin Resonance Investigations of Free Radicals Formed in Human Blood Components

**Figure 1:**
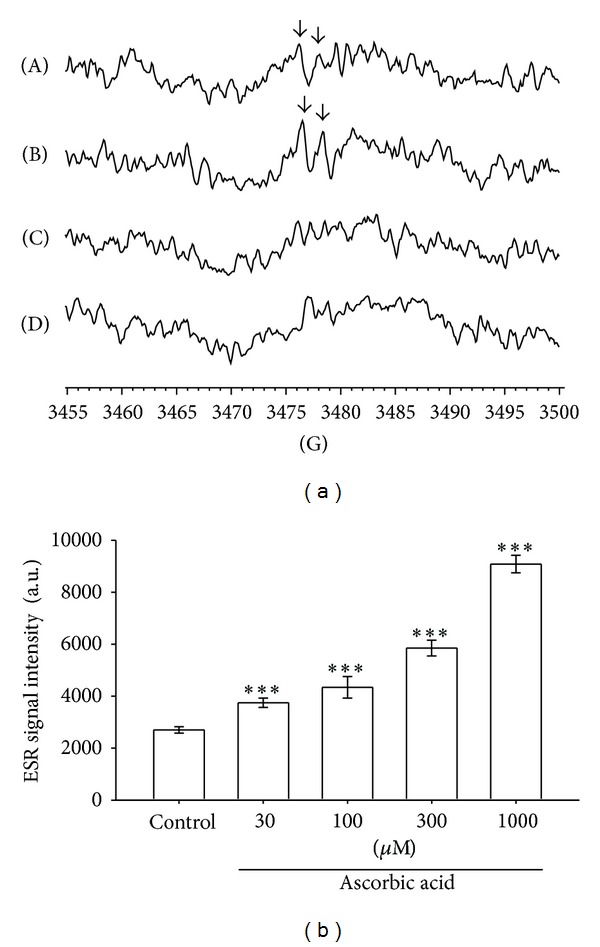
Free radical doublet (a) detected using ESR spectroscopy in (A) PPP, (B) PRP, (C) RBCs, and (D) whole blood. The ESR signal was examined at room temperature, and the following instrument parameters were used in ESR spectroscopy: standard frequency (X-band): 9 GHz; microwave power: 20 mW; modulation frequency: 100 kHz; time constant: 163.84 ms; conversion time: 40.96 ms; receiver gain: 5.02 × 10^5^; and the number of data X-scans: 4. The free radical doublet is marked with arrows: “↓”. Effect of ascorbic acid on the *g* = 2.00627 radical formation in human PRP. The intensity of the *g* = 2.00627 radical obtained from the reaction of PRP (approximately 8 × 10^6^ cells/mL, control) and 30 *μ*M, 100 *μ*M, 300 *μ*M, and 1000 *μ*M ascorbic acid in the presence of 100 mM DMPO. ESR analysis was exactly 30 s after the final addition. ESR spectra are labeled to show their components: DMPO- Asc^∙^ adduct (∗). The values of the ESR signal intensity in the bar chart (b) are shown as the means ± SEM (*n* = 4). ***P < 0.001 compared with the control. The instrument parameters were identical to those shown in (a).

Free radical signals were detected using ESR in human PPP, PRP, RBCs, and whole blood. A doublet signal radical was observed in PPP and PRP, but not in RBCs or whole blood (Figure 1(a)). PRP exhibited the strongest signal among the human blood components and was used in subsequent experiments. The hyperfine splitting and *g*-factor of this doublet signal were 1.88 G and 2.00627, respectively. In each instance, the signals exhibited doublet peaks and a line width of approximately 4 G. The radical species was identified to be ascorbyl based on the close similarity of the hyperfine coupling constants and *g*-factor of the observed signal to those of published data [13, 14]. No notable oxygen-derived free radicals were detected in this study, probably because of the presence of ascorbic acid and other antioxidants in human PRP.

### 3.2. Effect of Exogenous Ascorbic Acid on the *g* = 2.00627 Radical Formation in Human Platelet-Rich Plasma

To confirm that the *g* = 2.00627 radical was a typical Asc^∙^, we added exogenous ascorbic acid to human PRP. The intensity of the *g* = 2.00627 radical induced by exogenous ascorbic acid increased dose dependently (Figure 1(b)).

### 3.3. Effect of Superoxide and the Nitric Oxide Scavenger on Ascorbyl Free Radical Formation in Human Platelet-Rich Plasma

**Figure 2:**
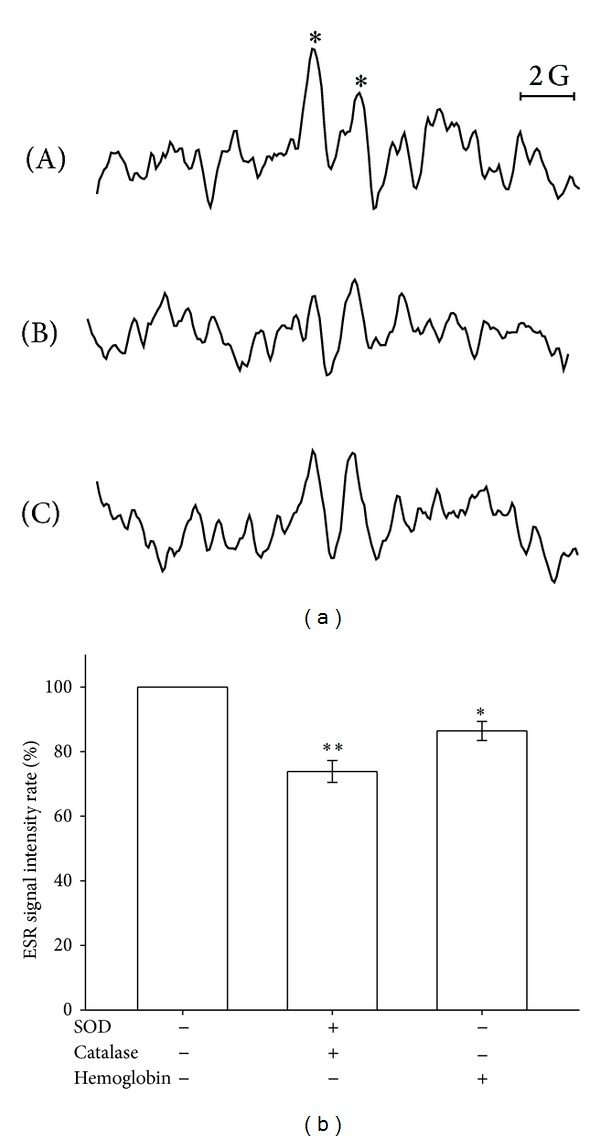
Effect of superoxide and the nitric oxide scavenger on Asc^∙^ formation in human PRP. ESR spectra (a) obtained from the reaction of (A) PRP (approximately 8 × 10^6^ cells/mL) and (B) superoxide scavenger (120 U/mL SOD and 1000 U/mL CAT) and the (C) nitric oxide scavenger (1 *μ*g/mL of hemoglobin) in the presence of 100 mM DMPO for 3 min. The ESR spectra are labeled to show their components: DMPO- Asc^∙^ adduct (∗). The ESR signal intensity rates in the bar chart (b) are shown as the means ± SEM (*n* > 3). **P < 0.01, *P < 0.05 compared with the control. The instrument parameters were identical to those shown in Figure 1(a).

We propose that the Asc^∙^ is a secondary radical; therefore, we determined which types of primary radical may be involved in the formation of this radical species. The effects of superoxide and the nitric oxide scavenger were examined on the *g* = 2.00627 radical formation, as shown in Figure 2. The *g* = 2.00627 signal formed by PRP was arbitrarily designated 100% and was inhibited by the superoxide scavenger (120 U/mL of SOD and 1000 U/mL of CAT) and nitric oxide scavenger (1 *μ*g/mL of hemoglobin) to 26.1% (*P* < 0.01) and 13.5% (*P* < 0.05), respectively. This result indicates that superoxide and nitric oxide may be primary radicals that induce Asc^∙^ formation.

### 3.4. Effect of the NADPH Oxidase Inhibitor on Ascorbyl Free Radical Formation in Human Platelet-Rich Plasma

**Figure 3:**
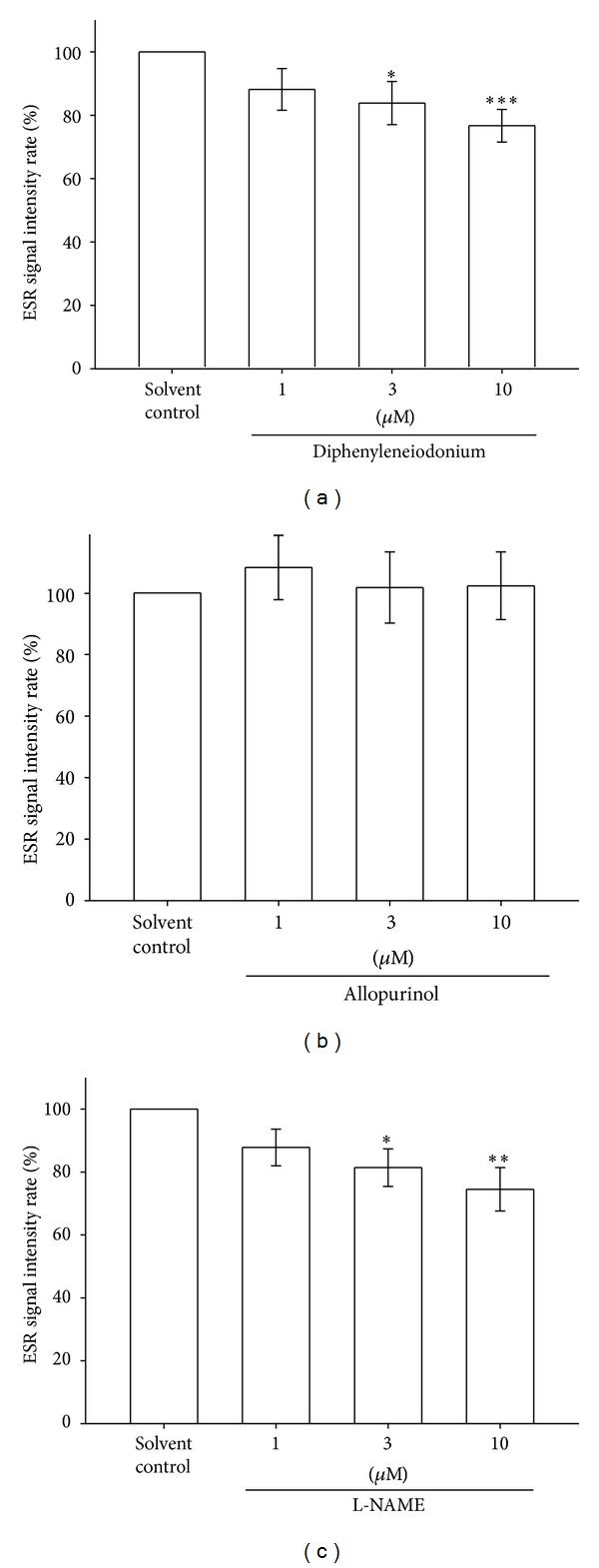
Effect of the NOX inhibitor (a), XO inhibitor (b), and NOS inhibitor (c) on Asc^∙^ formation in human PRP (approximately 8 × 10^6^ platelets/mL). The ESR signal intensity rates in the bar chart are expressed as the means ± SEM (*n*≧5). ***P < 0.001, *P < 0.05 compared with the solvent control. The instrument parameters were identical to those shown in Figure 1(a).

It was reported that NOX on the cell membrane of leucocytes may be the primary source of superoxide formation in blood [15]. To investigate the involvement of NOX in Asc^∙^ formation in PRP, we used DPI as a NOX nonselective inhibitor. The Asc^∙^ signal of a solvent control group was arbitrarily designated 100% and was dose dependently inhibited by DPI (3 *μ*M = 17.7%, *P* < 0.05; 10 *μ*M = 23.8%, *P* < 0.001, Figure 3(a)). This result indicates that NOX may be involved in the formation of Asc^∙^ in PRP.

### 3.5. Effect of the Xanthine Oxidase Inhibitor on Ascorbyl Free Radical Formation in Human Platelet-Rich Plasma

XO is a superoxide-producing enzyme normally present in the serum and lungs [16]. To investigate the involvement of XO in Asc^∙^ formation in PRP, we used allopurinol as a nonselective XO inhibitor. The Asc^∙^ signal of a solvent control group was arbitrarily designated 100%, and allopurinol (1–10 *μ*M) did not significantly influence Asc^∙^ formation in PRP (*P* > 0.05, Figure 3(b)).

### 3.6. Effect of the Nitric Oxide Synthase Inhibitor on Ascorbyl Free Radical Formation in Human Platelet-Rich Plasma

We determined whether NOS is involved in Asc^∙^ formation in PRP. We used L-NAME as an NOS inhibitor. The Asc^∙^ signal of a solvent control group was arbitrarily designated 100% and was inhibited by L-NAME (1–10 *μ*M) dose dependently (3 *μ*M = 18.6%, *P* < 0.05; 10 *μ*M = 25.4%, *P* < 0.01, Figure 3(c)). This result indicates that NOS-derived NO is associated with the formation of Asc^∙^ in PRP.

### 3.7. Effect of Arachidonic Acid on Ascorbyl Free Radical Formation in Human Platelet-Rich Plasma

**Figure 4:**
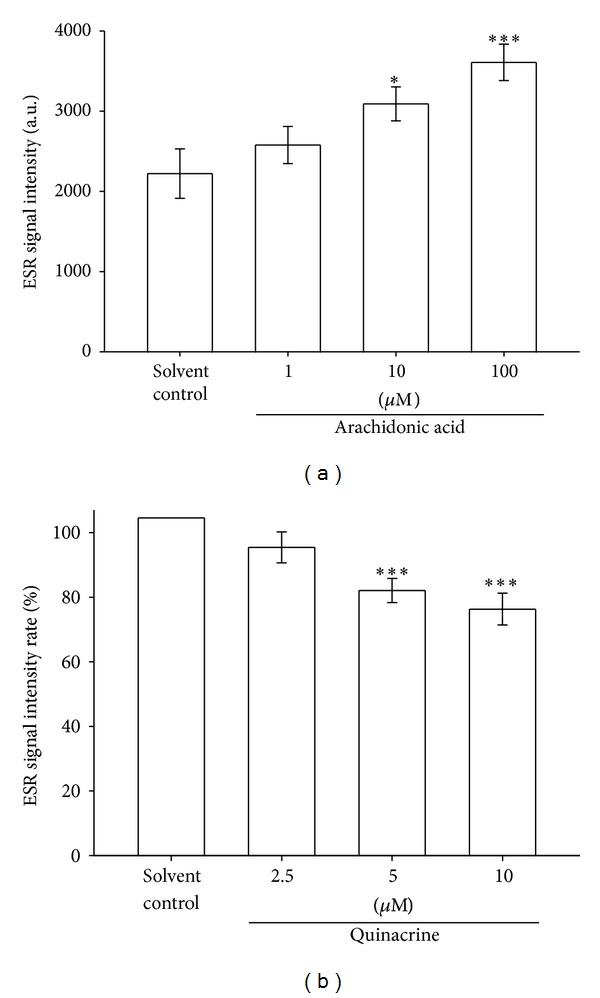
Effect of AA (a) and the PLA_2_ inhibitor (b) on Asc^∙^ formation in human PRP (approximately 8 × 10^6^ platelets/mL). The ESR signal intensity and data in the bar chart are expressed as the means ± SEM (*n*≧5). *P < 0.05, ***P < 0.001 compared with the solvent control group. The instrument parameters were identical to those shown in Figure 1(a).

Reactive oxygen species (ROS) are generated by AA metabolites, which are released from the cell membrane. AA-induced ROS generation may occur through the oxidative metabolic processes induced by COX and LOX [11]. AA has also been reported to induce ROS formation through NOX [17, 18]. Our results showed that NOX may be involved in the formation of the Asc^∙^ in PRP (Figure 3(a)). Therefore, we determined whether AA metabolite pathways are associated with the Asc^∙^ formation. The Asc^∙^ signal formed by PRP was, respectively, increased 39.1% (*P* < 0.05) and 62.4% (*P* < 0.001) by 10 *μ*M and 100 *μ*M AA compared with a solvent control (Figure 4(a)). In addition, the Asc^∙^ signal of the solvent control group was arbitrarily designated 100% and was inhibited by quinacrine (2.5–10 *μ*M), a phospholipidase A_2_ (PLA_2_) inhibitor, dose dependently (5 *μ*M = 20.9%, *P* < 0.001; 10 *μ*M = 26.2%, *P* < 0.001, Figure 4(b)). This result indicates that AA metabolite pathways are associated with the formation of the Asc^∙^ in PRP.

### 3.8. Effect of the Cyclooxygenase Inhibitor on Ascorbyl Free Radical Formation in Human Platelet-Rich Plasma

**Figure 5:**
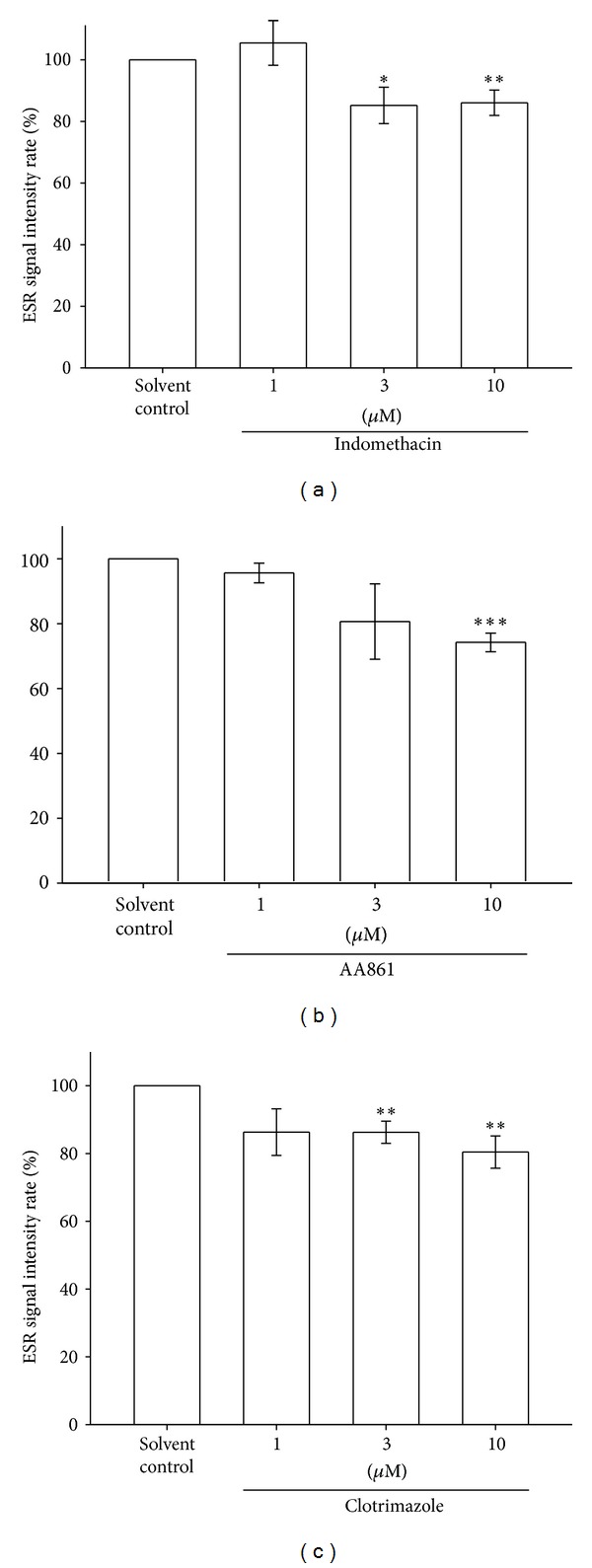
Effect of the COX inhibitor (a), LOX inhibitor (b), and CYP450 inhibitor (c) on Asc^∙^ formation in human PRP (approximately 8 × 10^6^ platelets/mL). The ESR spectra are labeled to show their components: DMPO- Asc^∙^ adduct (∗). The ESR signal intensity rates and data in the bar chart are expressed as the means ± SEM (*n*≧4). **P < 0.01, *P < 0.05 compared with the solvent control. The instrument parameters were identical to those shown in Figure 1(a).

In downstream pathways of the AA metabolism, COX [19], P450 [20], and LOX [21] are vital sources of extracellular ROS release. To investigate the involvement of COX in Asc^∙^ formation in PRP, we used indomethacin as a nonselective COX inhibitor. The Asc^∙^ signal of a solvent control group was arbitrarily designated 100%; 3 and 10 *μ*M indomethacin produced a 13.4% (*P* < 0.05) and 14.5% (*P* < 0.01) reduction of the Asc^∙^ signal, respectively. However, the signal was not significantly changed when a low dose (1 *μ*M) of indomethacin (Figure 5(a)) was used. This result suggests that COX may be involved in the formation of the Asc^∙^ in PRP.

### 3.9. Effect of the Lipoxygenase Inhibitor on Ascorbyl Free Radical Formation in Human Platelet-Rich Plasma

To investigate the involvement of LOX in Asc^∙^ formation in PRP, we used AA861 as a nonselective LOX inhibitor. The Asc^∙^ signal of a solvent control group was arbitrarily designated 100% and was inhibited by AA861 (1–10 *μ*M) dose dependently (10 *μ*M = 25.7%, *P* < 0.001, Figure 5(b)). This result suggests that LOX may also be involved in the formation of Asc^∙^ in PRP.

### 3.10. Effect of the P450 Inhibitor on Ascorbyl Free Radical Formation in Human Platelet-Rich Plasma

To investigate the involvement of P450 in Asc^∙^ formation in PRP, we used clotrimazole as a nonselective P450 inhibitor. As shown in Figure 5(c), 1 and 10 *μ*M clotrimazole produced 13.7% (*P* < 0.01) and 19.5% (*P* < 0.01) depressions of the Asc^∙^ signal, respectively.

### 3.11. Influence of the Mitochondrial Respiratory Chain on Ascorbyl Free Radical Formation in Human Platelet-Rich Plasma

**Figure 6:**
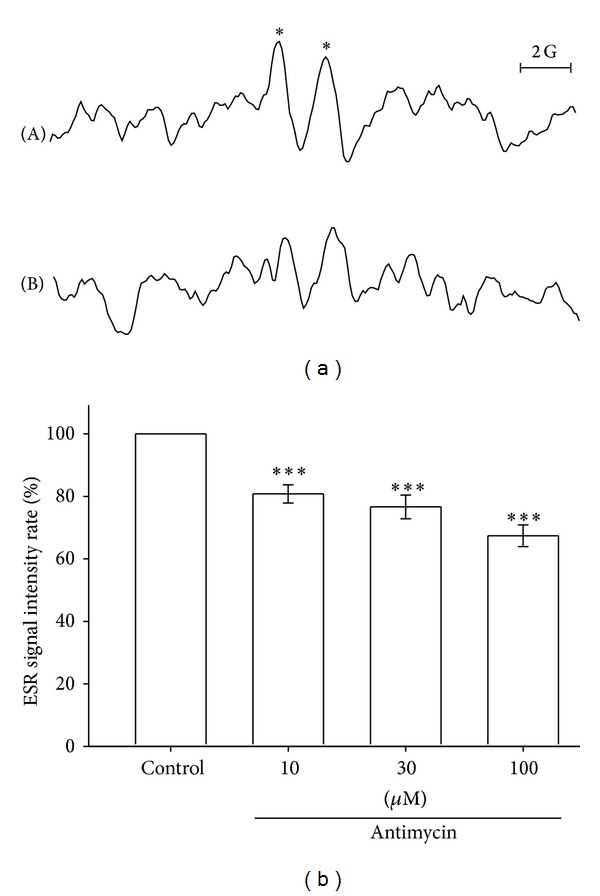
Effect of the mitochondrial complex III inhibitor on Asc^∙^ formation in human PRP. The ESR spectra show (a) the effect of a solvent control, (A) 0.6% DMSO, and 10, 30 (data not shown), and (B) 100 *μ*M antimycin in the presence of 100 mM DMPO for 30 min on Asc^∙^ formation in human PRP (approximately 8 × 10^6^ platelets/mL). The ESR spectra are labeled to show their components: DMPO- Asc^∙^ adduct (∗). The ESR signal intensity rate and data shown in the bar chart (b) are expressed as the means ± SEM (*n* = 6). ***P < 0.001 compared with the solvent control. The instrument parameters were identical to those shown in Figure 1.

In the mitochondrial respiratory chain, some electrons may leak to oxygen, partially reducing oxygen to a superoxide anion [22]. We determined whether oxidative stress induced by the mitochondrial respiratory chain is associated with Asc^∙^ formation in PRP. We used antimycin as a mitochondrial complex III inhibitor. The Asc^∙^ signal of a solvent control group was arbitrarily designated 100%, and 10, 30, and 100 *μ*M antimycin, respectively, produced 19.2% (*P* < 0.001), 23.3% (*P* < 0.001), and 32.5% (*P* < 0.001) depressions of the Asc^∙^ signal (Figure 6). This indicates that mitochondrial respiratory chain oxidative stress plays a partial role in Asc^∙^ formation in PRP.

### 3.12. Antioxidative Assay of Enzyme Inhibitors

**Figure 7:**
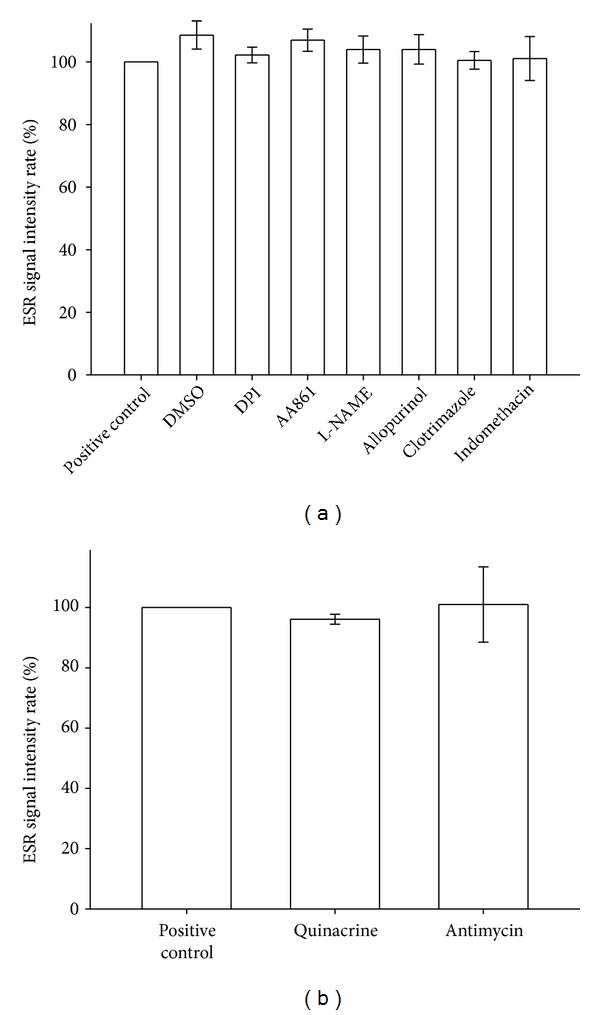
Effect of the fat-soluble (a) and water-soluble (b) enzyme inhibitor on hydroxyl free radical formation in the Fenton reaction. (a) The effect of the Fenton reaction solution (500 *μ*M hydrogen peroxide + 50 *μ*M FeSO_4_, positive control), 0.6% DMSO, 10 *μ*M DPI, 10 *μ*M AA861, 10 *μ*M L-NAME, 10 *μ*M allopurinol, 10 *μ*M clotrimazole, and 10 *μ*M indomethacin in the presence of 150 mM DMPO for 3 min on hydroxyl radical formation. (b) The effect of the Fenton reaction solution (positive control), 10 *μ*M quinacrine, and 100 *μ*M antimycin in the presence of 150 mM DMPO for 3 min on hydroxyl radical formation. The signal intensity rates and data shown in the bar chart are expressed as the means ± SEM (*n*≧3). *P* > 0.05 compared with the solvent control. The instrument parameters were identical to those shown in Figure 1(a).

Higashi et al. demonstrated that nordihydroguaiaretic acid, AA-861, and baicalein are LOX inhibitors and also have antioxidant activity [23]. However, Pallast et al. showed that AA-861 inhibits both 12/15-LOX and 5-LOX but does not have antioxidant activity [24]. Therefore, we selected AA-861 as a LOX inhibitor in this study. However, some enzyme inhibitors used in this study still potentially elicit antioxidative effects and inhibit Asc^∙^ signal production. To exclude this possibility, we used the Fenton reaction assay to determine whether the enzyme inhibitors were also antioxidants. The enzyme inhibitors were divided into lipid-soluble (Figure 7(a)) and water-soluble (Figure 7(b)) groups. Our result showed that DPI (10 *μ*M), AA861 (10 *μ*M), L-NAME (10 *μ*M), allopurinol (10 *μ*M), clotrimazole (10 *μ*M), indomethacin (10 *μ*M), quinacrine (10 *μ*M), and antimycin (10 *μ*M) did not exhibit significant antioxidative activity in the Fenton reaction assay (*P* > 0.05).

## 4. Discussion

The vascular endothelium plays an essential role in regulating vascular tone, modulating vascular growth, platelet aggregation and coagulation, and inflammation. Therefore, the degree of endothelial dysfunction may predict the outcomes of cardiovascular diseases [25]. Although the precise mechanisms of endothelial dysfunction have not been elucidated, a considerable amount of evidence suggests that increased oxidative stress may play a critical role in this state [26]. Oxidative stress can be described as an “imbalance between proxidants and antioxidants in favor of the proxidants, potentially leading to damage” [27]. Currently, reducing oxidative stress remains a prominent objective for cardiovascular prevention and therapy. However, clear knowledge of its source is required to provide novel perspectives for treatment.

ROS participate in the growth, apoptosis, and migration of vascular smooth muscle cells and in the remodeling of the vessel wall. Each of these responses may contribute to vascular diseases in uncontrolled conditions [28]. Therefore, the sources of ROS may be crucial therapeutic targets of cardiovascular disease.

In this study, we found that Asc^∙^ signals were observed in PPP and PRP, but not in RBCs or whole blood (Figure 1(a)). A study of ESR spectra of whole blood from normal and tumour bearing patients showed two main lines with *g* values of 4.2 and 2.049 [29]. The authors suggested that the line at *g* = 2.049 may be due to the copper protein ceruloplasmin. In addition, smaller signals were found with *g* values of 2.16, 2.005, and 1.98. We suggest that some paramagnetic species in whole blood with *g* values nearby 2.0 that restrict the signal intensity of ascorbyl radical. Therefore, in this study, we used ESR spectroscopy in detecting the Asc^∙^ to determine the sources of oxidative stress in human PRP. Asc^∙^ formation may be induced by nearly all ROS intrinsic to the biological environment, including superoxide radicals, hydroxyl radicals, alkyl peroxyl radicals, lipid peroxyl radicals, peroxynitrite, thiyl radicals, protein radicals, and catalytic metals [7].

**Figure 8:**
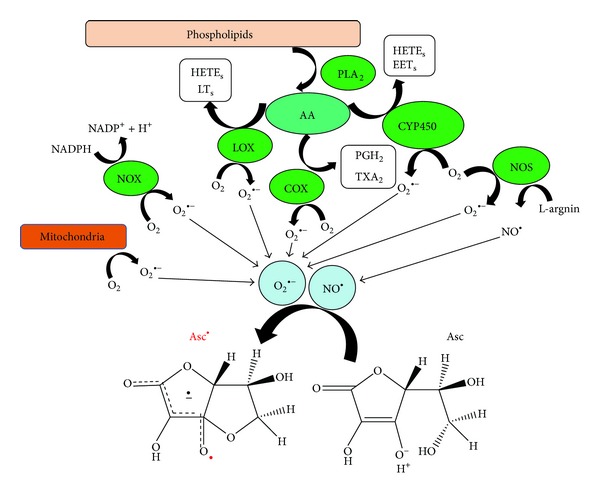
Proposed pathway for the mechanisms of Asc^∙^ formation in human PRP (O_2_^∙−^; superoxide anion; NO; nitric oxide).

In the vasculature wall, ROS are produced by all of the layers, and the major vascular ROS is the superoxide anion, which inactivates NO and, thus, impairs relaxation [30]. Superoxide-generating enzymes involved in increased oxidative stress within vascular tissue include uncoupled NOS, NOX, XO, and mitochondrial superoxide-generating enzymes [31]. In this study, we observed that the AA pathway enzymes, such as COX, LOX, and CYP450, also contributed to the increased oxidative stress in human PRP. However, the XO did not seem to play an important role in this event (Figure 8).

XO is capable of generating superoxide and hydrogen peroxide when supplied with its substrates, xanthine and hypoxanthine, which accumulate during ischemia [32]. Although studies have shown that XO is present in human arterial and venous endothelial cells and can generate sufficient levels of oxygen radicals to trigger endothelial injury, questions remain regarding the role of xanthine and hypoxanthine formation in triggering this process. Because the samples in this study were not subjected to ischemic conditions, we did not observe XO contributing to oxidative stress in human PRP.

NO is released from endothelial cells mainly by eNOS and is a main mediator of endothelium-dependent vasodilatation. When ROS production is increased, tetrahydrobiopterin generation is reduced, causing eNOS to uncouple and produce superoxide; when NO is insufficiently formed or quenched too quickly, the process of atherosclerosis is initiated or accelerated [33]. In pathological conditions, NO may be scavenged by excess ROS generated in blood vessels by vascular NOX [15]. eNOS has been observed not only in the endothelium but also in platelets [34]. Therefore, based on our results, we suggest that platelet eNOS is also a source of ROS in human PRP.

We recently applied ESR spectroscopy in detecting Asc^∙^ to investigate the oxidative status of lymphedema, suggesting that COX-derived oxidative stress plays a major role in the pathological mechanisms of surgically induced lymphedema [10]. However, in the current study, COX-derived oxidative stress played only a minor role in oxidative stress in human PRP.

## 5. Conclusion

In this study, we investigated the potential sources of Asc^∙^ production that contribute to oxidative stress in human PRP. We provide evidence that no single source of Asc^∙^ can be identified in human PRP, but Asc^∙^ are typically generated through NOX, COX, LOX, CYP450, eNOS, and mitochondrial superoxide-generating enzyme pathways.
